# Different treatment regimens in breast cancer visceral crisis: A retrospective cohort study

**DOI:** 10.3389/fonc.2022.1048781

**Published:** 2022-10-18

**Authors:** Ruohan Yang, Guanyu Lu, Zheng Lv, Lin Jia, Jiuwei Cui

**Affiliations:** Cancer Center, The First Hospital of Jilin University, Changchun, China

**Keywords:** breast cancer, visceral crisis, CDK4/6 inhibitors, targeted therapy, prognosis

## Abstract

**Objective:**

Breast cancer visceral crisis (VC) is caused by excessive tumor burden leading to severe organ dysfunction with poor prognosis. Traditional chemotherapy reduces the quality of life of patients without significantly improving survival. The aim of this study was to investigate the clinical characteristics of patients with VC and the prognosis by using different treatment options.

**Methods:**

According to the 5th European School of Oncology (ESO)–European Society for Medical Oncology (ESMO) international consensus guidelines for advanced breast cancer guidelines (ABC 5), patients who were treated in the First Hospital of Jilin University from 2018 to 2022 and diagnosed with breast cancer VC were retrospectively analyzed. The analysis focused on the characteristics of the patients, the treatment regimens, and prognosis.

**Results:**

A total of 133 patients were included in this study. As for metastasis breast cancer subtype, 92 (69.18%) were hormone receptor (HR) positive, human epidermal growth factor receptor 2 (HER-2) negative, 20 (15.04%) had HER-2 overexpression, and 21 (15.78%) were triple negative. All patients had an mOS of 11.2 months (range, 1.1–107.8 months). In different types of VC, the median overall survival (mOS) of bone marrow metastasis (BMM) was 18.0 months (range, 2.0–107.8 months), that of diffuse liver metastasis (DLM) was 8.1 months (range, 1.3–30.2 months), and that of meningeal metastasis (MM) was 9.0 months (range, 1.2–53.8 months). In 92 HR+, Her-2− patients using different treatment regimens, mOS was 6.2 months (range, 1.2–29.8 months) in the chemotherapy group while it was 24.3 months (range, 3.1–107.8 months) in the endocrine therapy (ET) group. Multivariate Cox regression analysis suggested that Eastern Cooperative Oncology Group (ECOG) scores and type of VC were associated with survival.

**Conclusion:**

Prognosis varied in different types of VC. Patients with BMM had the best prognosis, and DLM had the worst. As treatment options continue to progress, our retrospective study showed a significant prolongation of overall survival (OS) in patients with VC compared to previous studies.

## 1 Introduction

Global Cancer Observatory (GCO) reported that 684,996 people died of breast cancer worldwide in 2020, which is the second most common cause of death in female cancer patients (1). Metastatic breast cancer (mBC) is an incurable disease, which has prolonged the survival time of patients with mBC in recent years with the understanding of molecular typing of breast cancer (2, 3). For HR+ HER2− mBC, international guidelines recommend hormone therapy rather than chemotherapy, except for patients with visceral crisis (VC) (4, 5). Advanced breast cancer guidelines (ABC 5) defined VC as a severe organ dysfunction, with not only visceral metastasis but also concomitant vital organ damage (4). There are several main manifestations: (1) pulmonary lymphangitis with dyspnea, (2) bone marrow metastasis with hematopoietic dysfunction, (3) diffuse liver metastasis with liver function impairment, and (4) meningeal metastasis with meningeal irritation sign and superior vena cava syndrome caused by cervical lymph node compression (2, 6). Patients have mostly atypical clinical manifestations and may present with dyspnea, abdominal distension, pancytopenia, and headache (7–10). VC has a poor prognosis with an overall survival (OS) of only 3.7 months (2). About 70% of VC patients were hormone receptor (HR) positive (2, 11). However, their OS was not significantly longer in patients receiving chemotherapy compared with palliative care. Not only that, chemotherapy also reduces the patient’s life quality (6). The treatment of patients with HER-2 overexpression and triple-negative VC has not been reported.

Currently, cyclin-dependent kinase (CDK) 4/6 inhibitor combined with aromatase inhibitor (AI) or Fulvestrant prolongs survival in HR-positive visceral metastatic (non-VC) breast cancer patients. Turner et al. reported combination of CDK4/6 inhibitors with endocrine therapy (ET) for breast cancer visceral metastases, a median progression-free survival (mPFS) of 19.3 months and an objective response rate of 55% (12). Giovanna et al. reported a patient with VC treated with a letrozole in combination with palbociclib and leuprolide in complete remission for 23 months. Rahmat et al. also reported a VC patient treated with AI with an OS of 7 months (13). Anti-HER-2 therapies such as trastuzumab, pertuzumab, and antibody-conjugated drugs (ADCs) have also similarly prolonged survival in breast cancer patients (14). There is a lack of large-scale studies on the VC patients’ prognosis of treatment regimen other than chemotherapy.

Therefore, we conducted a retrospective study collecting the cases from our center to analyze the clinical characteristics of patients with VC of breast cancer and investigate the prognosis of patients using endocrine therapy, targeted therapy, and chemotherapy.

## 2 Method

### 2.1 Study population

Through the medical record system of the First Hospital of Jilin University from January 2018 to January 2022, a total of 733 patients with advanced breast cancer were included. Of them, there were 133 diagnosed with VC. The following characteristics of patients were retrospectively analyzed: (1) DLM: aspartate aminotransferase (AST) or alanine aminotransferase(ALT) > 3 times normal with or without total bilirubin (TB) > 1.5 times normal. (2) BMM confirmed by bone marrow aspirate biopsy. (3) MM usually suggests tumor cell infiltration by lumbar puncture or meningeal disease by brain MRI. (4) Pulmonary lymphangitis (PL) confirmed by lung computed tomography and fingertip blood oxygen inhalation SpO_2_ < 93% without oxygen inhalation. (5) Superior vena cava (SVC) syndrome presented with acute dyspnea and swelling face.

### 2.2 Data collection

Clinicopathologic information was abstracted by a review of medical records and included the following variables: HR (estrogen and progesterone) status, HER2 status, age at diagnosis of VC, Eastern Cooperative Oncology Group (ECOG) scores at diagnosis VC, treatment regimen received, time to disease progression, and time to patient death.

ET following diagnosis of VC includes AI, Fulvestrant, and CDK 4/6 inhibitor.

Adverse events (AEs) are graded according to Common Terminology Criteria for Adverse Events (CTCAE) Version 5.0.

### 2.3 Statistical analysis

Kaplan–Meier analysis was used to assess patient survival, and the log-rank test was used to determine OS rates between treatment groups using different regimens. Progression free survival (PFS) was defined as the time to disease progression from the time of occurrence of VC receiving any regimen. OS was defined as the time from the onset of VC to death.

## 3 Results

### 3.1 Clinical characteristics of all patients

A total of 733 patients with advanced breast cancer, of whom 133 (18.14%) have VC, were included in this study. Median age was 48 years (range, 21–69 years). Among them, 67 patients (50.38%) had DLM, 33 patients (24.81%) had BMM, 21 patients (15.79%) had MM, 10 patients (7.52%) had PL, and 2 patients (1.50%) had SVC. Ninety-two (69.18%) were HR+ Her-2−, 20 (15.04%) had HER-2 overexpression, and 21 (15.78%) were triple-negative. We analyzed factors such as pathological type and Ki-67 index (**Table 1**).

**Table 1:** Clinical characteristics of all patients (*N* = 133).

Variable	*N* (%)
**Age, years**	
Median age	48 (21–69)
≤60	119 (89.39%)
>60	14 (10.61%)
**Menstrual Status**	
Post-menopause	37 (27.82%)
Premenopausal	96 (72.18%)
**Molecular Typing**	
HR+, Her-2−	92 (69.18%)
HER-2 overexpression	20 (15.04%)
Triple negative	21 (15.78%)
**Pathological Type**	
Invasive ductal carcinoma	123 (92.48%)
Invasive lobular carcinoma	8(6.02%)
Others	2 (1.5%)
**Ki-67 expression**	
≥15%	124 (93.23%)
<15%	9 (6.77%)
**Histologic Grade**	
I	4 (3.01%)
II	48 (36.09%)
III	81 (60.90%)
**Visceral Crisis Type**	
BMM	33 (24.81%)
DLM	67 (50.38%)
PL	10 (7.52%)
MM	21 (15.79%)
SVC	2 (1.50%)
**Treatment Regimen**	
**Anti-HER-2 therapy**	20 (15.04%)
Trastuzumab + Pertuzumab	17 (12.78%)
Antibody–Drug Conjugate	3 (2.26%)
**Chemotherapy**	82 (62.59%)
Paclitaxel	28 (21.05%)
Platinum	26 (19.55%)
Gemcitabine	25 (18.79%)
Eribulin	3 (2.26%)
**Endocrine therapy**	31 (23.31%)
AI	15 (11.28%)
CDK4/6 inhibitors+AI	15 (11.28%)
CDK4/6+ Fulvestrant	1 (0.75%)
**ECOG scores at diagnosis of visceral crisis**	
1	33 (24.81%)
2	41 (30.82%)
3	56 (42.11%)
4	3 (2.26%)

#### 3.1.1 Clinical characteristics of patients with BMM

There were 32 patients with BMM, with a median age of 49.5 years (range, 29–68 years), all patients presented with decreased hemoglobin, 3 (9.09%) presented with thrombocytopenia, and 2 (6.06%) presented with pancytopenia without obvious cause (**Supplementary Table S1**).

#### 3.1.2 Clinical characteristics of patients with DLM

Of the 67 patients who developed DLM, the median age was 46 years (range, 27–64 years). Only 42 patients (62.69%) had elevated transaminases, and 25 patients (27.31%) had elevated transaminases combined with elevated bilirubin, and the detailed clinical characteristics are shown in **Supplementary Table S2**.

#### 3.1.3 Clinical characteristics of MM patients

Twenty-one patients developed MM with a median age of 40 years (range, 21–65 years) and presented with persistent headache without obvious cause and blurred vision, and lumbar puncture cerebrospinal fluid culture revealed tumor cell infiltration (**Supplementary Table S3**).

#### 3.1.4 Clinical characteristics of patients with PL and SVC

The number of patients with the above types of VC was small in this study, including 10 patients with PL and 2 patients with SVC. With a median age of 55 years (range, 35–69 years), all patients had a ki67 index ≥15% (**Supplementary Table S4**).

### 3.2 Prognosis

**Figure 1:**
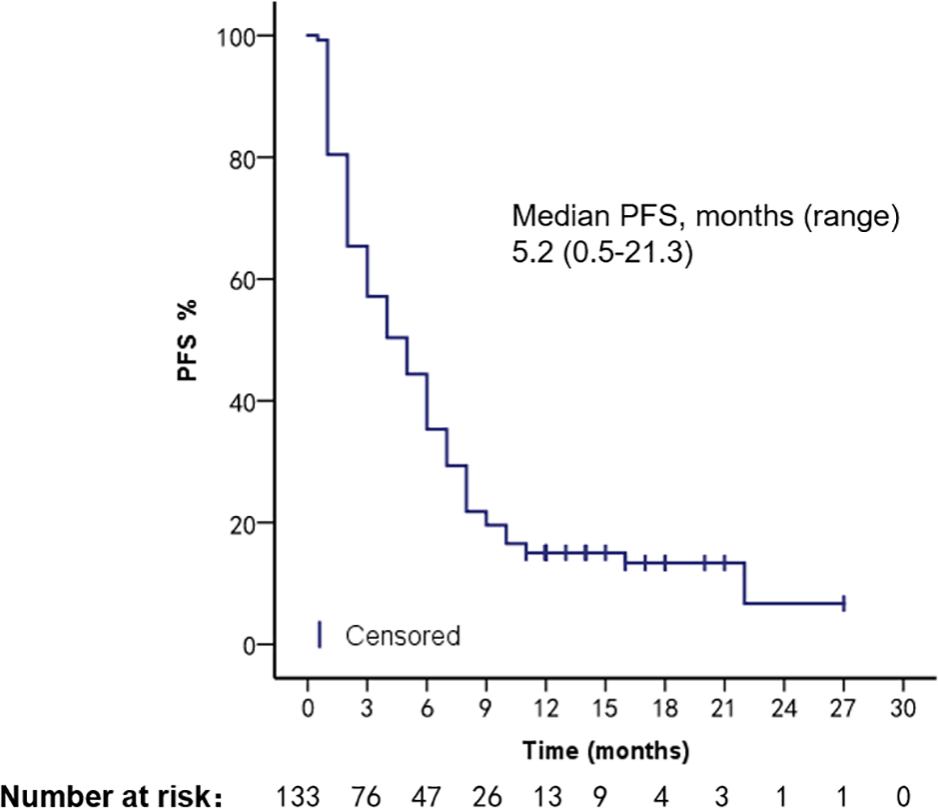
PFS of all VC patients.

**Figure 2:**
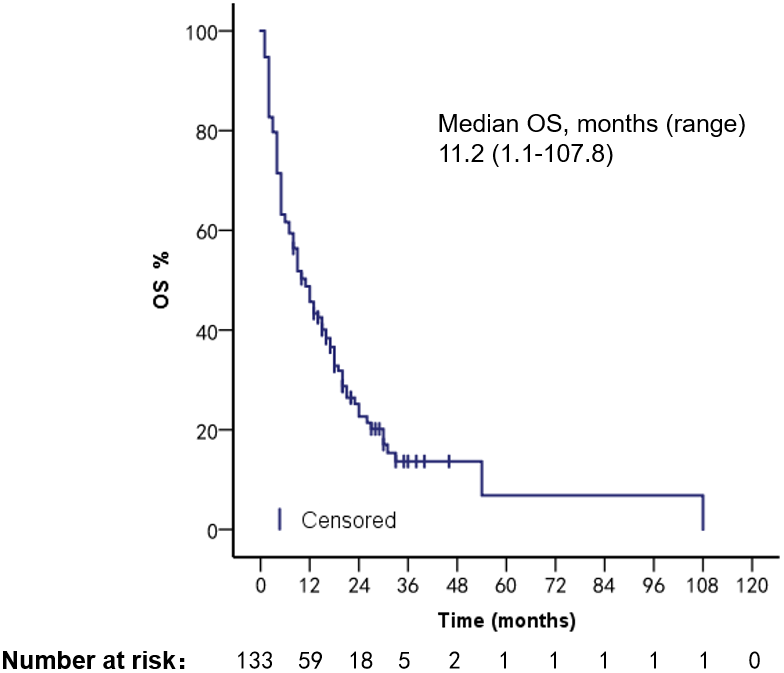
OS of all VC patients.

With a median follow-up time of 12 months (range, 1–110 months), 133 patients had a median overall survival (mOS) of 11.2 months (range, 1.1–107.8 months) and an mPFS of 5.2 months (range, 0.5–21.3 months) (**Figures 1**, **2**). We compared patient outcomes according to the following subgroups: type of VC, treatment regimen, and ECOG scores.

**Figure 3:**
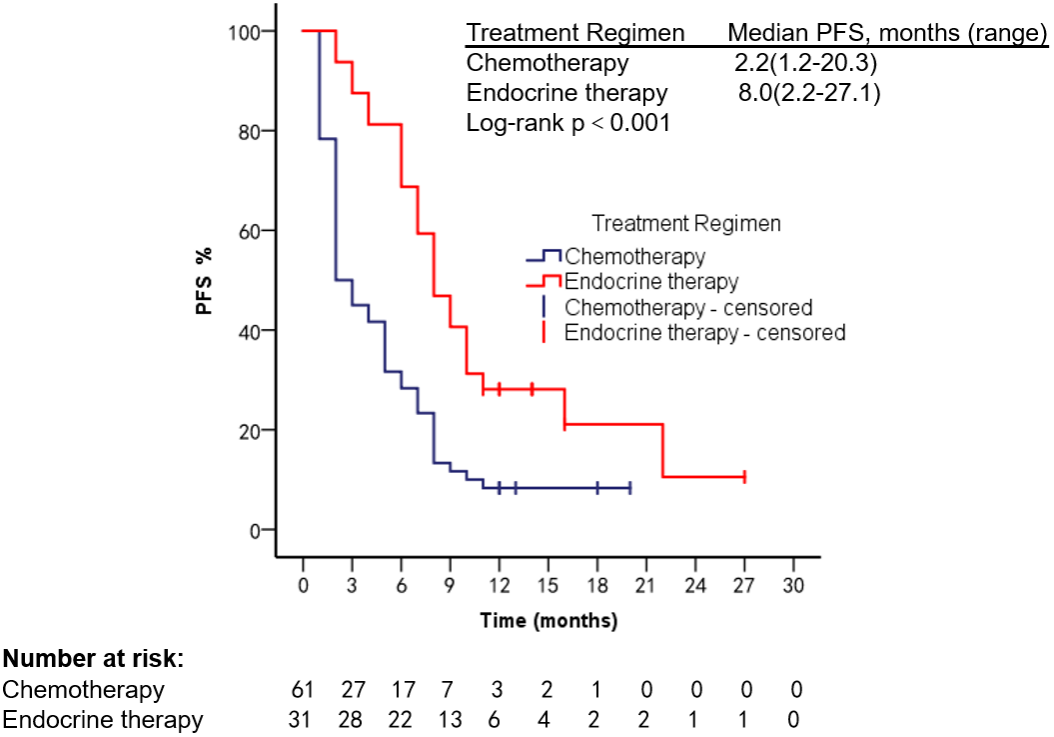
PFS of patients with different types of VC.

**Figure 4:**
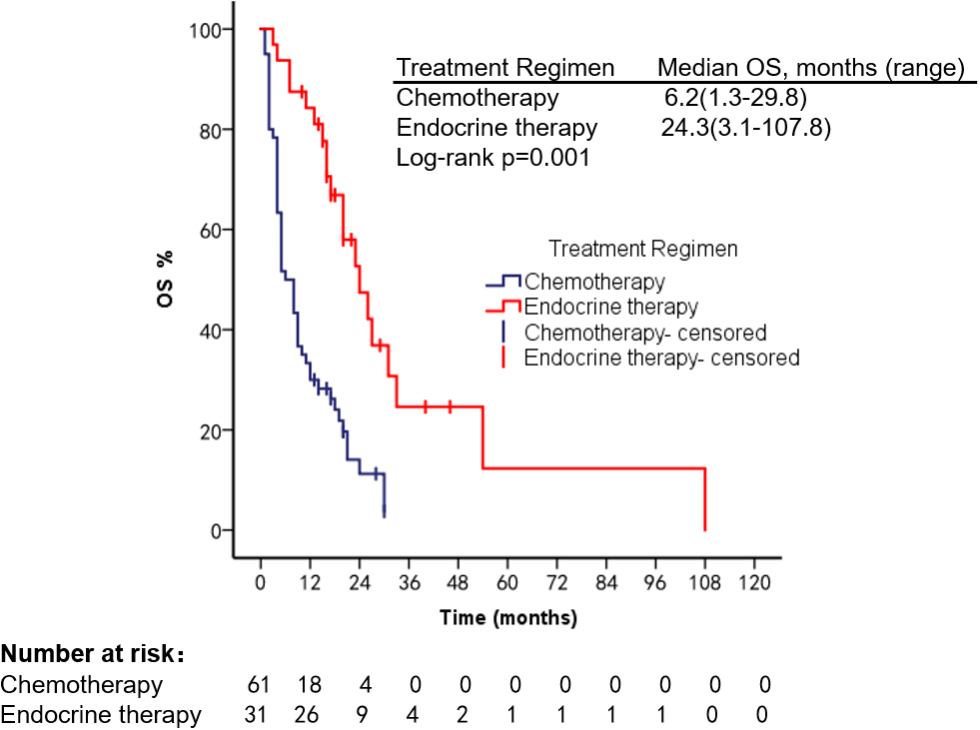
OS of patients with different types of VC.

In different types of VC, mPFS was 7.0 months (range, 1.0–22.0 months) in patients with BMM, 3.2 months (range, 0.5–14.3 months) in patients with DLM, and 3.3 months (range, 1.2–20.3 months) in patients with MM (Log-rank *p* = 0.005). mOS was 18.0 months (range, 2.0–107.8 months) in BMM patients, 8.1 months (range, 1.3–30.2 months) in patients with DLM, and 9.0 months (range, 1.2–53.8 months) in patients with MM (Log-rank *p* = 0.026) (**Figures 3**, **4**). The number of PL and SVC patients was too small to subgroup analysis.

**Figure 5:**
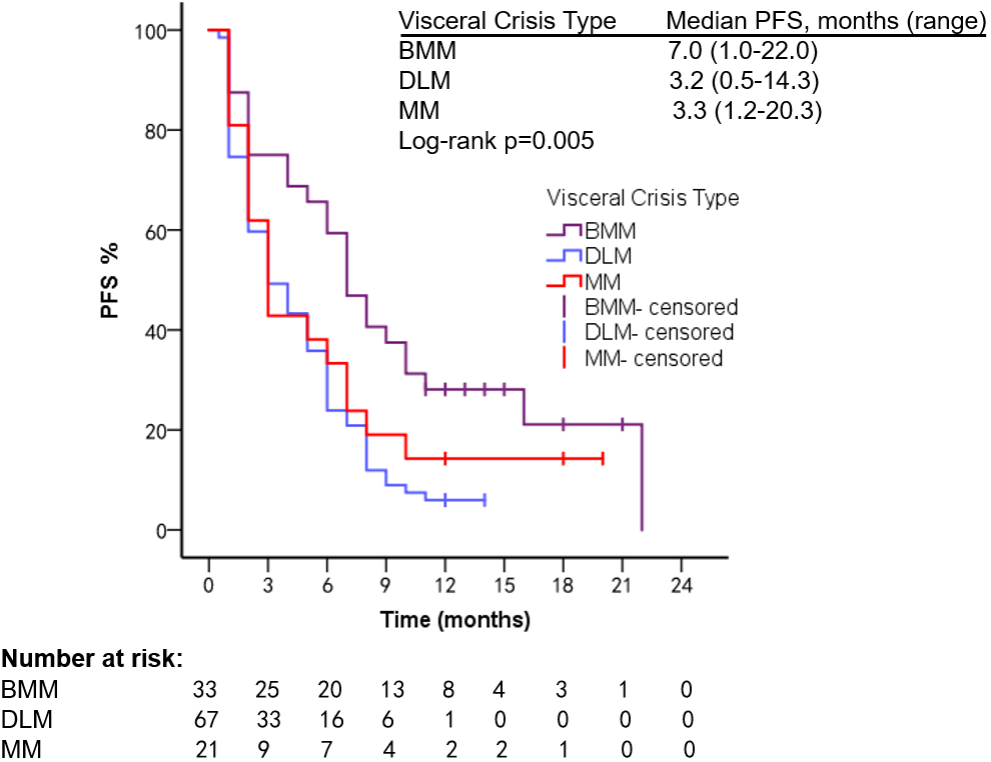
PFS of HR+, Her-2− patients after using different treatment regimens.

**Figure 6:**
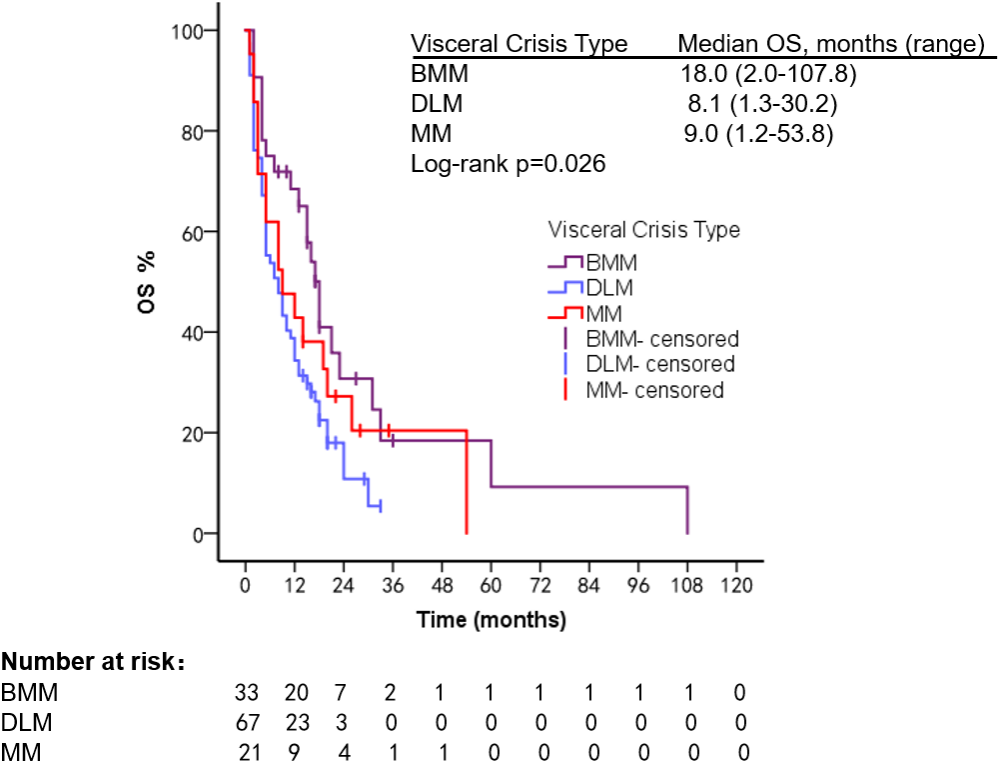
OS of patients with HR+, Her-2− type using different treatment regimens.

**Figure 7:**
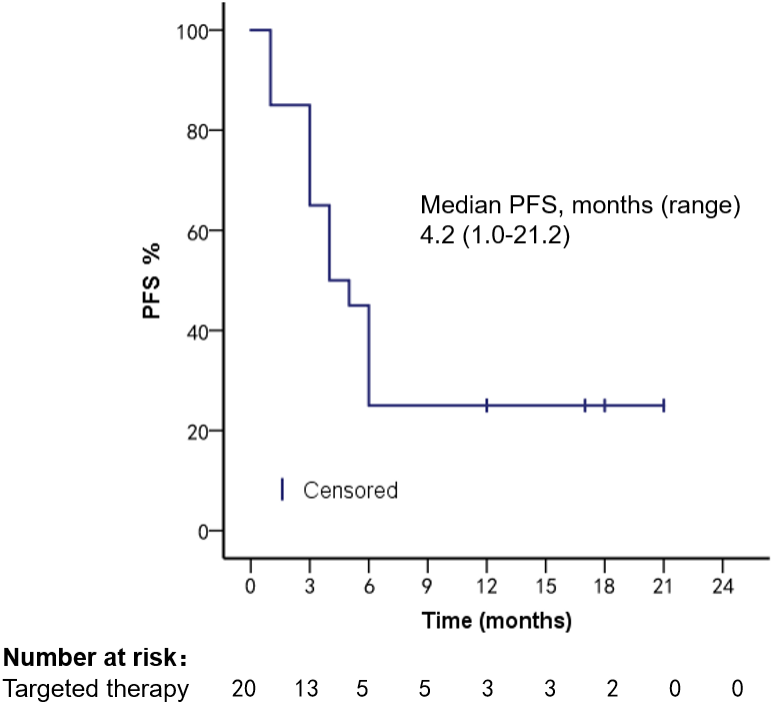
PFS in patients with Her-2 overexpression after targeted therapy.

**Figure 8:**
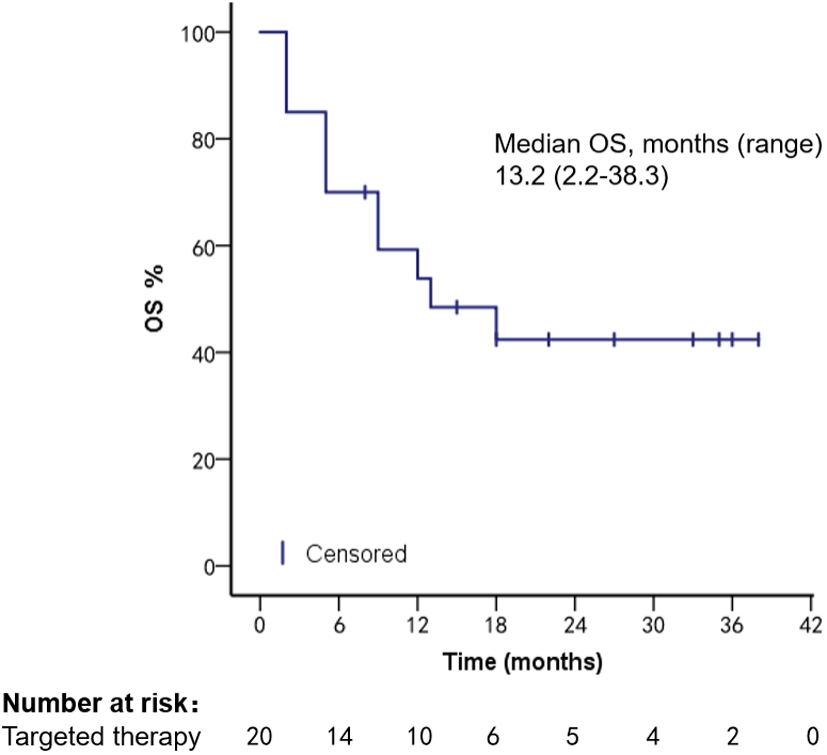
OS of Her-2 overexpressed patients after targeted therapy.

Survival analysis was performed in 92 HR+, Her-2− patients treated with different regimens; mOS was 6.2 months (range, 1.3–29.8 months) in the chemotherapy group, 24.3 months (range, 3.1–107.8 months) in the endocrine therapy (ET) group, mPFS 2.2 months (range, 1.2–20.3 months) in the chemotherapy group, and 8 months (range, 2.2–27.1 months, Log-rank *p* < 0.001) in the ET group. Targeted therapy was used in all 20 Her-2 overexpression patients; mPFS was 4.2 months (range, 1.0–21.2 months) and mOS was 13.2 months (range, 2.2–38.3 months), as shown in **Figures 5**–**8**.

**Figure 9:**
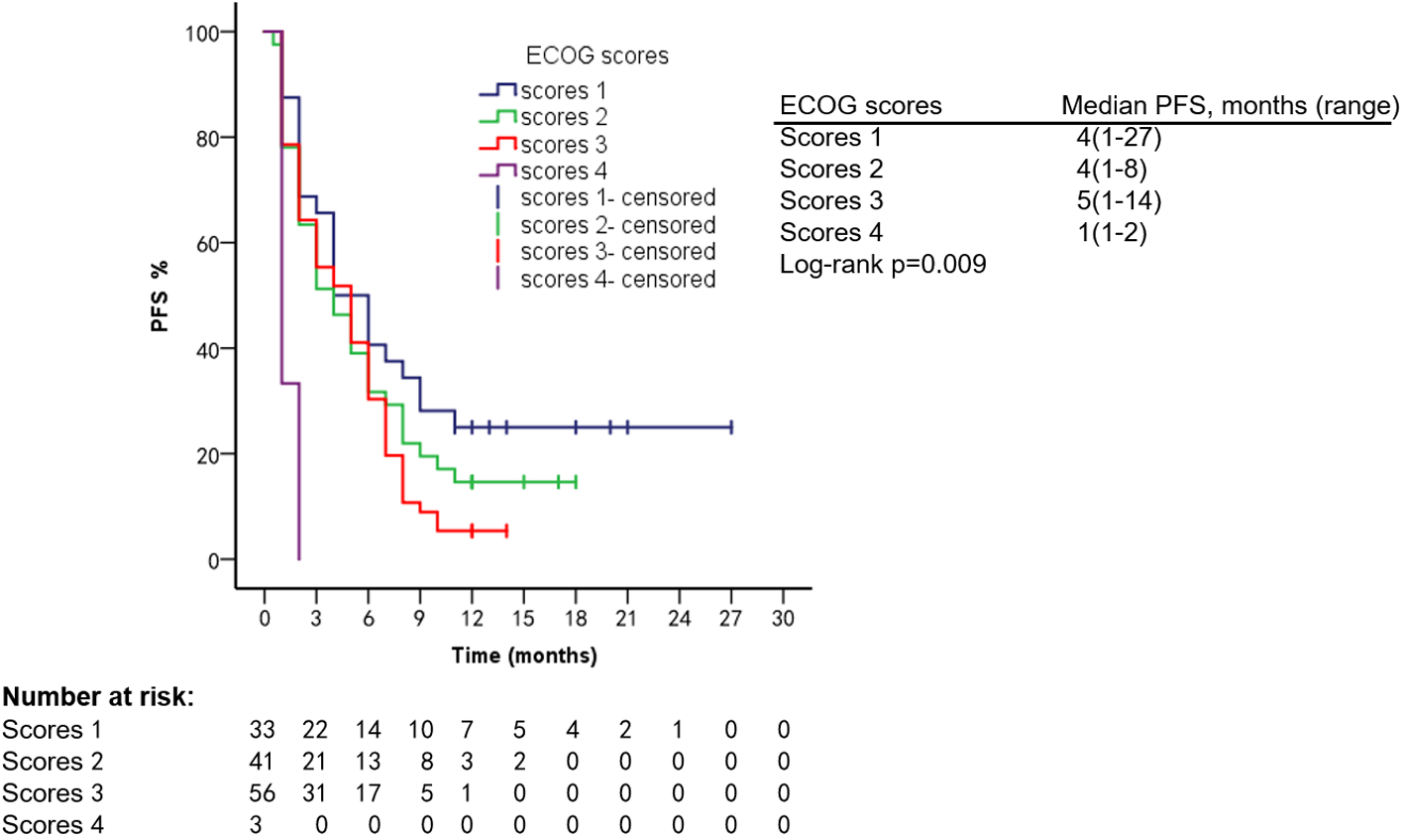
PFS of patients with different ECOG scores.

**Figure 10:**
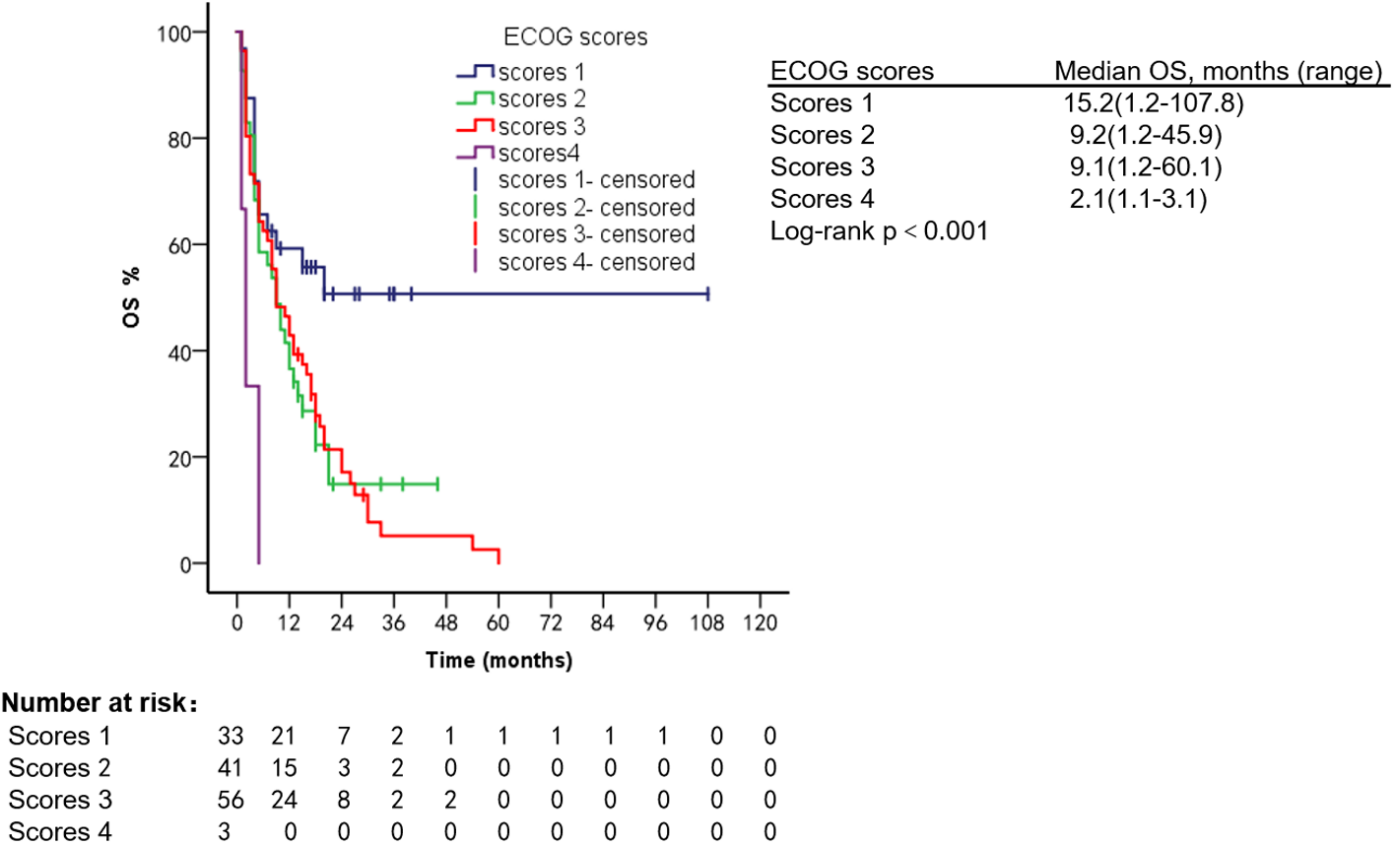
OS of patients with different ECOG scores.

Patients had different ECOG scores at the time of diagnosis of VC and different prognoses. Patients with an ECOG score of 1 had an mPFS of 4.1 months (range, 1.2–27.1 months) and an mOS of 15.2 months (range, 1.2–107.8 months); ECOG score 2: mPFS was 4.1 months (range, 1.1–8.1 months) and mOS was 9.2 months (range, 1.2–45.9 months); ECOG score 3: mPFS was 5.1 months (range, 1.1–14.2 months) and mOS was 9.1 months (range, 1.2–60.1 months); ECOG score 4: mPFS was 1.1 months (range, 1.1–1.9 months) and mOS was 2.1 months (range, 1.1–3.1 months) (**Figures 9**, **10**).

To investigate factors influencing the prognosis of patients with VC, we performed Cox proportional hazards models including age, menstrual status, histological grade, ki-67 index, and multiple factors and found that ECOG performance status scores (95% CI: 1.29–2.25, *p* = 0.016) and type of VC (95% CI: 1.03–1.31, *p* = 0.02) were associated with survival (**Table 2**).

**Table 2:** Multivariate Cox regression analysis.

Factors	*Number*, %	Median OS, months (95% CI)	Hazard Ratio value (95% CI)	*p*-value
**Age**			1.059 (0.48-2.2)	0.934
≤60	119, 89.39%	10 (10.98–16.20)		
>60	14, 10.61%	7.5 (4.36–18.63)		
**Menstrual status**			0.682 (0.36–1.27)	0.227
Post-menopause	37, 27.82%	8 (7.77–20.87)		
Premenopausal	96, 72.18%	12 (11.29–15.98)		
**Molecular typing**			1.056 (0.88–1.27)	0.560
HR+, Her-2−	92, 69.18%	11 (11.05–18.32)		
HER-2 overexpression	20, 15.03%	13 (10.24–21.08)		
Triple negative	21, 15.15%	3.5 (3.54–9.05)		
**Ki-67 expression**			1.406 (0.58-3.37)	0.445
≥15%	124, 93.23%	9 (10.41–14.57)		
<15%	9, 6.77%	15 (9.38–49.72)		
**Histological grading**			1.443 (1.07–1.98)	0.177
I	4, 3.01%	10 (–6.5–35.5)		
II	48, 36.09%	11 (10.60–16.20)		
III	81, 60.90%	9 (9.69–16.90)		
**ECOG scores**			1.443 (1.07–1.98)	0.016
1	33, 24.81%	15 (10.26–24.73)		
2	41, 30.82%	9 (8.05–14.43)		
3	56, 42.11%	9 (9.84–16.44)		
4	3, 2.26%	2 (2.5–7.83)		
**Pathological type**			0.894 (0.43–1.85)	0.764
Invasive ductal carcinoma	123, 92.48%	9 (10.58–14.69)		
Invasive lobular carcinoma	8, 6.02%	18 (11.22–25.89)		
Others	2, 1.5%	Not Available		
**Visceral crisis type**			1.102 (1.03–1.31)	0.020
BMM	33, 24.81%	14 (2.31–48.98)		
DLM	67, 50.38%	8 (7.99–11.94)		
PL	10, 7.52%	9 (7.85–20.05)		

### 3.3 Adverse events

Adverse events (AEs) are summarized in **Table 3**. The most common hematologic AEs in the chemotherapy group was neutropenia (85.36%), with grade ≥ 3 AEs in 14 (17.07%) patients, and alopecia (100%) was the most common non-hematologic AEs. The most common hematologic AE in the targeted therapy group was neutropenia (6 patients, 30%); nausea and vomiting (7 patients, 35%) were the most common non-hematologic AEs; the most common hematologic AE in the ET group was also neutropenia (19.35%), and fatigue was the most common non-hematologic AE (28.81%). Twenty-six (31.70%) patients in the chemotherapy group, five (25%) in targeted therapy group, and five (16.13%) in the ET group had dose reductions due to AEs. No patients experienced treatment-related serious adverse events (SAEs).

**Table 3:** AEs after treatment.

	Chemotherapy (*n* = 82)	Targeted Therapy (*n* = 20)	Endocrine Therapy (*n* = 31)
	All Grades Grade ≥ 3	All Grades Grade ≥ 3	All Grades Grade ≥ 3
**Hematologic AEs**			
Neutropenia	70 (85.36%) 14 (17.07%)	6 (30%) 1 (5%)	6 (19.35%) 0
Leukopenia	48 (58.54%) 9 (10.98%)	3 (15%) 0	3 (9.68%) 1 (3.23%)
Hemoglobin decreased	56 (68.29%) 11 (13.41%)	2 (10%) 0	5 (16.13%) 1 (3.23%)
Thrombocytopenia	21 (25.61%) 13 (15.85%)	0 0	3 (9.68%) 0
**Non-hematologic AEs**			
Alopecia	82 (100) 0	2 (10%) 0	0
Fatigue	63 (76.83%) 5 (6.10%)	6 (30%) 0	8 (28.81%) 0
Nausea and vomiting	34 (41.46%) 4 (4.88%)	7 (35%) 1 (5%)	0
Neurotoxicity	18 (21.95%) 3 (3.66%)	0	0
**AEs results in dose reduction**	26 (31.70%)	5 (25%)	5 (16.13%)

## 4 Discussion

About 70%–80% of breast cancer patients with early stage are curable (15); however, about 20%–30% of them will develop distant metastasis, and a proportion of them will develop VC due to excessive tumor burden (2, 16). The incidence rate of VC was not counted in the previous study. We found that between 2018 and 2022, VC occurred in 18.14% of 733 patients with advanced disease who were treated at the First Hospital of Jilin University and diagnosed with breast cancer VC. DLM was the most common VC type (50.38%), which is consistent with the findings of Maria et al. (**Supplementary Table S5**) (2).

Yassir et al. found a median age of 48 years in patients with VC (6). This is similar to our findings (49.5 years). We found that the majority (118 cases, 89.39%) of patients with VC were older than 60 years, invasive ductal carcinoma was the most common pathological type (123 cases, 93.18%), and HR+, Her-2− type was the most common molecular type (92cases, 69.18%), which was consistent with the reported literature (2, 11).

We performed a subgroup analysis of the clinical features of patients with different types of VC, and 33 patients developed BMM, all of whom presented with non-treatment-related anemia and fatigue; 84.38% had high Ki-67 expression, which was similar to the clinical features of 30 patients with BMM of breast cancer reported by Abdullah et al. (**Supplementary Table S5**). However, the present study found that 75.75% of patients with bone marrow metastases were accompanied by pathogenically negative fever, with a higher incidence than that reported by Li Xiao et al., which suggests to us that when breast cancer patients present with anemia and fever with negative etiological test, BMM should be taken into account. We found that patients with DLM had varying degrees of transaminase elevations and marked elevations in bilirubin. Molecular typing was also prevalent with HR+, Her-2− (68.68%). The clinical characteristics were consistent with Estela et al., who summarized 30 published cases (17). Patients with MM in our study presented with headache and blurred vision, which were associated with neurological symptoms due to cancer cell invasion of the leptomeninges, arachnoid membrane, and subarachnoid space (18); our patient was clinically consistent with the reported literature (19–21). PL patients presented with progressive dyspnea with decreased partial pressure of oxygen; this is consistent with Monika’s study (7). The mechanism may be that tumor cells spread along lymphatic vessels and pulmonary interstitium and prevent blood gas exchange in the lung (22).

Despite aggressive chemotherapy, the prognosis of patients with VC remains poor and patients die within a short period of time due to disease progression (2, 6). It has been reported that the most benefit in survival time is presented by Chikako et al. who retrospectively analyzed 44 patients with VC receiving paclitaxel combined with bevacizumab chemotherapy, with an mPFS of about 4 months and an mOS of about 10 months (11). This is consistent with our VC patients (**Supplementary Table S5**).

We analyzed the prognosis of different types of VC and found that patients with BMM had the best prognosis with an mOS of 18.0 months, a longer survival time than the currently reported studies (23, 24). Patients with DLM had the worst prognosis, with an mOS of 8.1 months. However, Estela et al. reported DLM survival of less than 1 week (17). A pilot study by Ricky et al. found that mOS could reach 6.5 months using vinorelbine combined with cisplatin for DLM (**Supplementary Table S5**) (25). Survival of our DLM patients was also superior to the reported studies. We found that MM patients had an mOS of 9 months. Anna et al. retrospectively analyzed patients with breast cancer MM and found that mOS was about 4.3 months (19). Our survival was significantly better than previous studies, which may be related to patients receiving different treatment regimens. Monika et al.’s meta-analysis of 24 breast cancer patients with PL found that despite aggressive chemotherapy, patients had an mOS of only 20 days (7), while Jean-David et al. reported a case of chemotherapy with eribulin in 2018 with an mOS that could reach 50 months (26). This suggests that new microtubule inhibitors may allow patients to achieve longer survival. The number of patients who developed PL and SVC was small and no survival analysis was performed.

In recent years, the use of CDK4/6 inhibitors has prolonged the survival of patients with HR-positive mBC (27). A single-center prospective clinical trial was conducted by Technische University Munchen in 2020 (Identifier: NCT04681768) to explore the efficacy of CDK4/6 combined with AI in patients with high-burden mBC; no relevant results have been published. We found that PFS and OS were significantly longer in HR+, Her-2− patients treated with ET than in the chemotherapy group (*p* < 0.001). We also found that patients who underwent ET reached a maximum PFS of 27 months, with no progression by the end of follow-up. The longest OS was 107.8 months, longer than OS in the reported studies. For patients with HER-2 overexpressing mBC, the use of trastuzumab, pertuzumab, and ADC drugs can improve patient outcomes (14, 28). Xu Long et al. reported an OS of 11 months in a patient with VC treated with trastuzumab (29). Anti-Her-2 therapy was also found to prolong survival in our study. We analyzed factors that may influence the prognosis of breast cancer patients with VC, and found that higher ECOG scores were associated with shorter survival, which is consistent with reported results (2). The type of VC was also a factor affecting patients’ prognosis (95% CI: 1.03–1.31, *p* = 0.02).

We counted AEs occurring with different treatment regimens, and granulocytopenia was the most common in the chemotherapy group (**Table 3**), which was similar to the findings of Chikako et al. (11). The proportion of AEs was less in the targeted therapy and ET groups than in the chemotherapy group. AE results in dose reduction were also lowest in the ET group. Therefore, safer and more effective treatment options in addition to chemotherapy can be selected for patients with VC who have fair physical performance.

VC with organ dysfunction, excessive tumor load, and rapid disease progression limit the application of drugs. The prognosis is poor. The guidelines do not include CDK4/6 inhibitors combined with AI as standard treatment, probably because of the rapid disease progression and the slow onset of ET. However, our study found that HR+, Her-2− patients treated with ET showed good safety and efficacy. In clinical work, the use of CDK4/6 inhibitors combined with AI may be an option for individualized treatment of patients with VC.

## 5 Conclusion

Among breast cancer patients with different types of VC, BMM had the best prognosis, while DLM had the worst prognosis. Compared with conventional chemotherapy, the use of CDK4/6 inhibitors combined with AI and anti-HER-2 therapy can significantly prolong VC patients’ survival and improve quality of life.

## Data availability statement

The original contributions presented in the study are included in the article/**Supplementary Material**. Further inquiries can be directed to the corresponding authors.

## Ethics statement

Ethical review and approval was not required for the study on human participants in accordance with the local legislation and institutional requirements. Written informed consent for participation was not required for this study in accordance with the national legislation and the institutional requirements. Written informed consent was not obtained from the individual(s) for the publication of any potentially identifiable images or data included in this article.

## Author contributions

RY: Writing—original draft; review and editing. GL: Writing—review and editing. ZL: Writing—review and editing. LJ: Methodology, funding acquisition, and writing—review and editing. JC: Conceptualization, methodology, supervision, and writing—original draft; review and editing. All authors contributed to the article and approved the submitted version.

## Funding

This research was funded by the National Natural Science Youth Foundation of China (Grant No. 82001670).

## Acknowledgments

We thank all the patients and the authors involved in this study.

## Conflict of interest

The authors declare that the research was conducted in the absence of any commercial or financial relationships that could be construed as a potential conflict of interest.

## Publisher’s note

All claims expressed in this article are solely those of the authors and do not necessarily represent those of their affiliated organizations, or those of the publisher, the editors and the reviewers. Any product that may be evaluated in this article, or claim that may be made by its manufacturer, is not guaranteed or endorsed by the publisher.
