# Supplementary material for: Different treatment regimens in breast cancer visceral crisis: A retrospective cohort study
Source: Front Oncol. 2022 Oct 18;12:1048781. doi: 10.3389/fonc.2022.1048781 (PMC9623315; doi:10.3389/fonc.2022.1048781)
Supplement: Supplementary file 1 [file DataSheet_1.docx]

**Supplementary Table S1** **Clinical features of patients with BMM:（n=33）**

| **Variable** | **N（%）** |
| --- | --- |
| **Age, years** |  |
| Median age | 49.5 （29-68） |
| ≤60 | 25 （75.75%） |
| ＞60 | 7 （24.25%） |
| **Menstrual status** |  |
| Post-Menopause | 11（33.33%） |
| Premenopausal | 22（66.67%） |
| **Molecular typing** |  |
| HR +, HER-2 - | 25（75.75%） |
| HER-2 overexpression | 3 （9.09%） |
| Triple negative | 5 （15.15%） |
| **Pathological type** |  |
| Invasive ductal carcinoma | 28（84.85%） |
| Invasive lobular carcinoma | 2 （6.06%） |
| Papillary carcinoma | 3 （9.09%） |
| **Ki-67 expression** |  |
| ≥15% | 28（84.85%） |
| ＜15% | 5 （15.15%） |
| **Histological grading** |  |
| I | 1 （3.03%） |
| II | 18（54.55%） |
| III | 14（42.42%） |
|  |  |
| **Clinical manifestations** |  |
| Fatigue | 33（100%） |
| Fever | 25（75.75%） |
| **Blood routine** |  |
| **Pancytopenia** | 2（6.06%） |
| **Decreased hemoglobin (g/L)** | 33（100%） |
| 100-80 | 18（54.55%） |
| ＜80 | 15（45.45%） |
| **Thrombocytopenia (10^9^/L)** | 4 （12.12%） |
| 75-99 | 3 （9.09%） |
| ＜75 | 1 （3.03%） |

**Supplementary Table S2** **Clinical features of patients with DLM（n=67）**

| **Variable** | **N（%）** |
| --- | --- |
| **Age, years** |  |
| Median age (range) | 46 （27-64） |
| ≤60 | 62 （92.53%） |
| ＞60 | 5 （7.47%） |
| **Menstrual status** |  |
| Post-menopause | 16（23.88%） |
| Premenopausal | 51（76.12%） |
| **Molecular typing** |  |
| HR +, HER-2 - | 42（62.68%） |
| HER-2 overexpression | 12（17.91%） |
| Triple negative | 13（19.41%） |
| **Pathological type** |  |
| Invasive ductal carcinoma | 63（94.03%） |
| Invasive lobular carcinoma | 3 （4.48%） |
| Others | 1 （1.49%） |
| **Ki-67** **expression** |  |
| ≥15% | 64（95.52%） |
| ＜15% | 3 （4.48%） |
| **Histological grading** |  |
| I | 2 （2.99%） |
| II | 19（28.36%） |
| III | 46（68.66%） |
| **Liver function** |  |
| Elevated transaminases | 42（62.69%） |
| Elevated transaminases with elevated total bilirubin | 25（37.31%） |

**Supplementary Table S3** **Clinical features of patients with MM（n=21）：**

| **Variable** | **N（%）** |
| --- | --- |
| **Age, years** |  |
| Median age (range) | （21-65） |
| ≤60 | 20 (95.24%) |
| ＞60 | 1 (4.76%) |
| **Menstrual status** |  |
| Post-menopause | 3 （14.29%） |
| Premenopausal | 18（85.71%） |
| **Molecular typing** |  |
| HR +, HER-2 - | 16（76.19%） |
| HER-2 overexpression | 3 （14.29%） |
| Triple negative | 2 （9.52%） |
| **Pathological type** |  |
| Invasive ductal carcinoma | 20（95.23%） |
| Invasive lobular carcinoma | 1 （4.77%） |
| **Ki-67 expression** |  |
| ≥15% | 20（95.23%） |
| ＜15% | 1 （4.77%） |
| **Histological grading** |  |
| I | 1 （4.77%） |
| II | 7 （33.33%） |
| III | 13（61.90%） |

**Supplementary Table S4** **Clinical characteristics of patients with PL (n=10) and SVC（n=2）**

| **Variable** | **N（%）** |
| --- | --- |
| **Age, years** |  |
| Median age (range) | 55 （35-69） |
| ≤60 | 8 （66.66%） |
| ＞60 | 4 （33.34%） |
| **Menstrual status** |  |
| Post-menopause | 7（58.33%） |
| Premenopausal | 5（41.67%） |
| **Molecular typing** |  |
| HR +, HER-2 - | 9 （75%） |
| HER-2 overexpression | 2 （16.7%） |
| Triple negative | 1 （8.3%） |
| **Pathological type** |  |
| Invasive ductal carcinoma | 12（100%） |
| Invasive lobular carcinoma | 0 （0%） |
| **Ki-67 expression** |  |
| ≥15% | 12（100%） |
| ＜15% | 0 （0%） |
| **Histological grading** |  |
| I | 0 （0%） |
| II | 4 （33.33%） |
| III | 8 （66.67%） |

**Supplementary Table S5. Summary of clinical characteristics and prognosis of reported breast cancer patients with visceral crisis.**

| Type of Study | Research content | Sample size | Median age | Molecular typing | PFS | OS | Result | References |
| --- | --- | --- | --- | --- | --- | --- | --- | --- |
| Retrospective study | Visceral crisis | 35 | 48 | Luminal A ：35 | —— | 4.7weeks | The management of patients with Luminal mBC and visceral crisis is a complex issue. Measured by short-term survival, the prognosis is poor. Chemotherapy has limited effect. | ^6^ |
| Retrospective study | Visceral crisis | 44 | 60 | Luminal A：35  Triple negative：9 | 131days（4.36） | 323days（10.76） | Paclitaxel combined with bevacizumab has achieved good efficacy and safety in the treatment of patients with visceral crisis, so it can be considered as an option for the treatment of visceral crisis. | ^11^ |
| Retrospective study | Visceral crisis | 261 | 54.5 | Luminal A：166 Luminal B：17  Her-2（+）：21  triple negative：57 | —— | 3.7months | Compared with patients without visceral crisis, patients with visceral crisis had significantly lower overall survival, and hyperbilirubinemia and poor ECOG performance status were associated with prognosis. | ^2^ |
| Retrospective study | Bone marrow metastasis | 30 | 44.5 | Luminal ：22  Her-2（+）：4  Triple negative：4 | —— | 9months | The application of chemotherapy can prolong the survival time of breast cancer with bone marrow metastasis. Of these regimens, paclitaxel treatment had the highest survival rates. | ^23^ |
| Retrospective study | Bone marrow metastasis | 22 | 47 | Luminal：18  Her-2（+）：1  Triple negative：3 | —— | 11months | Most patients have improved bone marrow function after chemotherapy and have the potential for long-term survival. | ^24^ |
| Review | Diffuse liver metastases | 32 | 47.9 | —— | —— | 16days | Absence of lesions on abdominal CT does not exclude diffuse liver metastases in a patient with acute liver failure (ALF) from breast cancer who died due to rapid disease progression. | ^17^ |
| Pilot study | Diffuse liver metastases | 11 | 60 | Estrogen receptor（+）：6  Progesterone receptor (+)：5  Her-2（+）：4 | —— | 6.5months | Vinorelbine combined with cisplatin in the treatment of patients with diffuse liver metastases can normalize liver function and provide the possibility to prolong survival. | ^25^ |
| Retrospective study | Meningeal metastasis | 318 | 54 | Luminal A：140  Luminal B+her-2（+）：83  Triple negative：81  Unknown：14 | —— | 3.5months | Most LM patients have limited survival. | ^20^ |
| Retrospective study | Meningeal metastasis | 187 | 49 | Luminal A：75  Luminal B：20  Her-2（+）：13  Triple negative：64  Unknown：15 | —— | 17weeks | In the entire LM patient group, factors associated with prolonged survival were: advanced age, high KPS score, breast cancer luminal subtype, systemic therapy, radiation therapy, and intrathecal therapy. | ^19^ |
| systematic review and meta-analysis | Lung cancer lymphangitis | 24 | 49.21 | —— | —— | 20days | PLC is a visceral crisis with a poor prognosis, but symptomatic and antitumor therapy should be considered. | ^7^ |
